# Zebrafish models of cancer: progress and future challenges^☆^

**DOI:** 10.1016/j.gde.2013.11.003

**Published:** 2014-02

**Authors:** Jennifer Yen, Richard M White, Derek L Stemple

**Affiliations:** 1 Wellcome Trust Sanger Institute, Wellcome Trust Genome Campus, Cambridge CB10 1SA, United Kingdom; 2 Memorial Sloan Kettering Cancer Center and Weill-Cornell Medical College, New York, NY 11788, United States

## Abstract

The need for scalable strategies to probe the biological consequences of candidate cancer genes has never been more pressing. The zebrafish, with its capacity for high-throughput transgenesis, *in vivo* imaging and chemical/genetic screening, has ideal features for undertaking this task. Unique biological insights from zebrafish have already led to the identification of novel oncogenic drivers and small molecules being used to treat the human cancer. This review summarizes the recent main findings and describes pertinent areas where the zebrafish can greatly contribute to our understanding of cancer biology and treatment.

**Current Opinion in Genetics & Development** 2014, **24**:38–45

This review comes from a themed issue on **Cancer genomics**

Edited by **David J Adams** and **Ultan McDermott**

For a complete overview see the Issue and the Editorial

Available online 27th December 2013

0959-437X/$ – see front matter, © 2013 The Authors. Published by Elsevier Ltd. All rights reserved.

**http://dx.doi.org/10.1016/j.gde.2013.11.003**

## Introduction

The wealth of genetic and transcriptomic data in cancer biology, accumulated through international cancer efforts such as The Cancer Genome Atlas (TCGA) and International Cancer Genome Consortium (ICGC), present unprecedented opportunities for identifying therapeutically meaningful targets. A major challenge in genomic approaches has been the lack of appropriate model systems in which to test these on a large scale. Owing to its small size, heavy brood, and rapid maturation time, the zebrafish has emerged as an important new cancer model that complements what can traditionally be achieved in mice and cell culture systems. Advances in transgenic and mutagenesis strategies have already led to a wide variety of zebrafish cancer models with distinct capabilities for high-throughput screening and *in vivo* imaging [1^•^,2,3^•^,4–16]. Despite significant progress in the past 10 years, however, the unique role of zebrafish in cancer research has still yet to be defined. Here, we review recent major achievements in the zebrafish cancer field in light of the available models and advances in genomic techniques. We conclude by discussing future areas of research where zebrafish efforts will be the most effective.

### Blood tumors

Numerous leukemic lines have been generated since the first zebrafish model of leukemia was reported in 2003, in a landmark paper showing that expression of mouse *c-Myc* in transgenic zebrafish unleashed rapid leukemia development [1^•^]. Consisting of a variety of T or B-cell lymphoblastic (ALL) and myeloid (AML) malignancies, zebrafish leukemia is typically modeled through the expression of a frequently mutated proto-oncogene (such as *c-Myc* [1^•^], *TEL-AML* [4] and *NOTCH1* [6]) under the *rag2* promoter in developing lymphocytes. A major advantage of this system is the tagging of a fluorescent marker to the gene of interest, enabling powerful real-time tracking of lymphocyte migration and proliferation.

An illustrative example of this tool is an elegant work by Feng *et al.*, in studying a *Bcl-2;Myc* zebrafish model of lymphoblastic lymphoma (T-LBL) [17]. In this study, Feng *et al.* monitored the local metastatic behavior of *Discosoma* red (ds-RED) tagged zebrafish lymphocytes in transparent *casper* fish, which had vasculature defined by enhanced green fluorescence protein (EGFP). Through live imaging of these cells, the authors were able to determine that lymphoblast autophagy was responsible for preventing their intravasion into the marrow, a hallmark transition of T-LBL to acute T-ALL. Cross-testing in zebrafish and human T-LBL cell lines revealed that this autophagy was caused by high levels of S1P1, which when suppressed resulted in widespread dissemination of the disease (Table 1).

In another study, live imaging of zebrafish embryos enabled Ridges *et al.* to identify a selective inhibitor of lymphocyte proliferation that is remarkably effective against human T-ALL xenografts [18^••^]. Ridges *et al.* screened over 26 000 chemicals for activity that could diminish fluorescent-tagged lymphocyte development in zebrafish larvae. One compound, lenaldekar, induced long-term remission in a zebrafish T-ALL model with encouraging responses in efficacy and toxicity when targeted against human xenografts in mice. While the drug's mechanism remains to be determined, this study provides a key example of the application of zebrafish for pre-clinical drug discovery.

In contrast to T-ALL, efforts to study acute myeloid leukemia (AML), the most lethal and commonly diagnosed leukemia, have not been as successful. To our knowledge, there is one zebrafish AML model and it is based on expression of the *MOZ/TIF2 (MYST3/NCOA2)* fusion gene under *spi1* control in the kidney, where hematopoiesis occurs in zebrafish [19]. Attempts to model AML from proto-oncogenes *KRAS*^G12D^ [20], *NUP98-HOXA9* [21] and *AML1-ETO* [22] have instead led to new models of myeloproliferative neoplasms (MPN) that for unknown reasons do not advance to AML. While the early MPN phenotypes provide valuable read-outs for chemical-genetic screening [23^•^], their inability to progress to AML may indicate biological differences in this system that warrant further investigation.

In spite of these and other exciting discoveries, there remain areas of active challenge in modeling leukemia in zebrafish. These include to what extent the models truly recapitulate basic aspects of the human disease, to what extent they can be used as models for interrogating genomic changes, and how they can be most effectively used to identify new drug targets across a wider range of disease types. In the coming years, large scale testing of candidate drivers (culled from the TCGA type efforts) in zebrafish leukemic lines will be necessary for these models to further demonstrate their worth.

### Solid tumors

Improved transgenic strategies have enhanced the complexity and diversity of solid tumor models in zebrafish, many of which were established through *N*-ethyl-*N*-nitrosourea (ENU) mutagenesis screens of mutations in specific genes of interest, such as the important tumor suppressor genes *tp53*, *apc* and *pten* [3^•^,5,24]. Here we focus on two rapidly growing areas of solid tumor model research: melanoma and embryonal rhabdomyosarcoma.

#### Melanoma

The first experimental confirmation that oncogenic *BRAF*^V600E^ (*BRAF)*, mutated in 40–50% of human melanomas [25–27], can promote nevi (moles) and melanoma formation was demonstrated in zebrafish [7]. Since then, similar findings have been shown with *NRAS*^Q61K^ [8] although this model remains less exploited thus far. The simplicity of visualizing melanoma development in these models has led to their widespread adoption and several important, proof-of-principle experiments.

Using the *BRAF* model, Ceol *et al.* [28^••^] tested the oncogenicity of 30 candidate melanoma cancer genes found in a region recurrently amplified in human metastatic melanoma [29]. Genes were overexpressed in melanocytes through the injection of a miniCoopR shuttle vector system into *BRAF* and *p53* mutant embryos. By monitoring for accelerated tumor onset, Ceol *et al*. were able to identify that *SETDB1*, a histone transferase, is an oncogene that causes more aggressive melanoma development in zebrafish. This work was the first to demonstrate the feasibility of high-throughput screening of candidate cancer genes in zebrafish and establishes a basis for guiding future approaches aiming to filter down large cancer datasets.

In another application of this line, *BRAF* expression was associated with a distinct gene signature that resembled expression profiles of embryonic neural crest stem/progenitor cells, thereby motivating White *et al.* [30^••^] to screen for suppressors of this embryonic phenotype. A class of compounds, called inhibitors of dihydroorotate dehydrogenase (DHODH), was found to selectively abrogate neural crest development in zebrafish as well as melanoma growth in mouse xenografts and human cell lines. Currently being followed in Phase I/II clinical trials, the DHODH inhibitor leflunomide is a pivotal demonstration of how an embryonic phenotype can be translated to findings about the human disease and lead molecules from zebrafish research into clinical investigation.

Detailed live imaging of melanocytes in a temperature sensitive *mitfa* (*mitfa*^vc7^) mutant has provided novel insights into the direct consequences of *mitfa* activity on tumorigenesis. Reduced *mitfa* activity caused a dramatic increase in melanocyte cell division [31] and was found to directly affect tumor morphology and formation in the *BRAF* model [32^•^]. As these findings could be reversed with the restoration of *mitfa*'s activity, this work substantiates the notion that *mitfa* is a modifier of *BRAF*-driven melanoma and provides a functional link between low *MITF* expression in patients with their poor melanoma prognosis.

#### Embryonal rhabdomyosarcoma

Recent studies using a *KRAS*^*G12D*^-driven model of embryonal rhabdomyosarcoma (ERMS) [11] have highlighted the importance of the cell of origin as a determinant of ERMS. For example, Ignatius *et al.* [33] used dynamic cellular imaging of a mosaic transgenic *rag2-KRAS*^*G12D*^ model to track the movement and evolution of ERMS cell subpopulations in embryonic and adult zebrafish. Their findings revealed new roles for differentiated ERMS cells in tumor growth and suggest that mechanisms governing their homeostatic maintenance in regulating growth could be relevant considerations in developing potential therapeutic treatment.

In a similar approach, using promoters representing various stages of muscle development (*cdh15*, *rag2*, *mylz2*), Storer *et al.* [34] drove expression of *KRAS*^G12D^ and observed that tumors that originated from the more progenitor like cells were more invasive and undifferentiated. These tumors were found to closely recapitulate subgroups of human ERMS based on differentiation status and harbor unique signaling pathways in each subgroup. Confirmation of these pathways as therapeutic targets awaits further study but demonstrates how cross-species oncogenomics can be used to guide therapeutic targeting strategies.

Important insights have also been described in other zebrafish models that cannot be described here [35–39] (reviewed in [40^••^,41^••^,42,43]). It is apparent though that some tumor types are better modeled in zebrafish than others. Major areas that have not been as well developed include reliable, penetrant models of pancreatic adenocarcinoma and intestinal carcinoma. While some attempts in this direction have been made [44], these and other diverse solid tumors will require further development.

### Comparative oncogenomic approaches

One of the biggest challenges in experimental cancer research is to demonstrate that the model in question recapitulates the human disease. While zebrafish tumors generally resemble their intended human cancers on a histological level [1^•^,7,8,24], there remain differences in tumor spectrum, incidence and onset [3^•^,5,24] that are still not well understood. An emerging mode of comparison is through new genomic technologies, which, with careful exploitation, may also point to genetic events that are important for malignant human tumor evolution.

Several studies have begun to compare genomic aberrations in zebrafish cancer to those in human. Rudner *et al.* [45] employed high-density array comparative genomic hybridization (aCGH) to zebrafish and human T-ALL and found a small number of repeatedly altered genes in zebrafish that also recur in human. Greater overlap was shown in samples from advanced stages of the disease, indicating a heightened conservation for genes under selective pressure. In another study, Zhang et al. [46] sequenced a large cohort of zebrafish malignant peripheral nerve sheath tumors (MPNSTs) and distinguished amplified genes that were shared with the human disease. While the identification of these commonly mutated genes is a promising first step, their experimental validation will be critical toward demonstrating their biological significance.

Our group recently investigated the full spectrum of coding mutations in a zebrafish cancer through exome sequencing of melanomas derived from *BRAF* and *NRAS*-driven transgenic lines [76]. In probing for secondary genetic events important for melanoma development, we found that the mutation burden in zebrafish melanomas was sparse compared to human cancer, and equally heterogeneous to the point that cross-species comparisons were difficult. Despite the mutation load, we were able to quantify the multi-hit model of these engineered cancers and highlight a potential new cooperating event with *BRAF* and *p53* mutation through the protein kinase A-cyclic AMP pathway. The work provides the first insights into the mutagenic processes of an engineered zebrafish cancer and will be instructive in guiding future studies of this type in zebrafish.

In particular, it is clear from our experience that there are technical challenges in adapting sequencing tools to zebrafish that require substantial optimization and development. The tremendous diversity both within and between zebrafish strains [47,48], nearly a magnitude greater than that of human, combined with the duplicated genome and other species-specific differences can complicate alignment and overwhelm somatic mutation algorithms with false calls. For this reason, extensive confirmation of these mutations is paramount to avoid errors and to ensure that the data are suitable for meaningful analyses.

While these issues are being addressed, genomic pursuits in zebrafish can focus on modalities that are more robust to nuances in alignment, such as genomic copy number changes and transcriptome profiles based on RNA-seq. The latter strategy provides the additional advantage of capturing a wider range of aberrations — important given the heterogeneity — that together converge on a single expression phenotype. This and optimization of available tools will provide researchers far greater scope for evaluating the relevance of zebrafish cancer and in prescribing new targets and strategies for investigating the human disease.

### Future prospects and challenges

**Figure 1:**
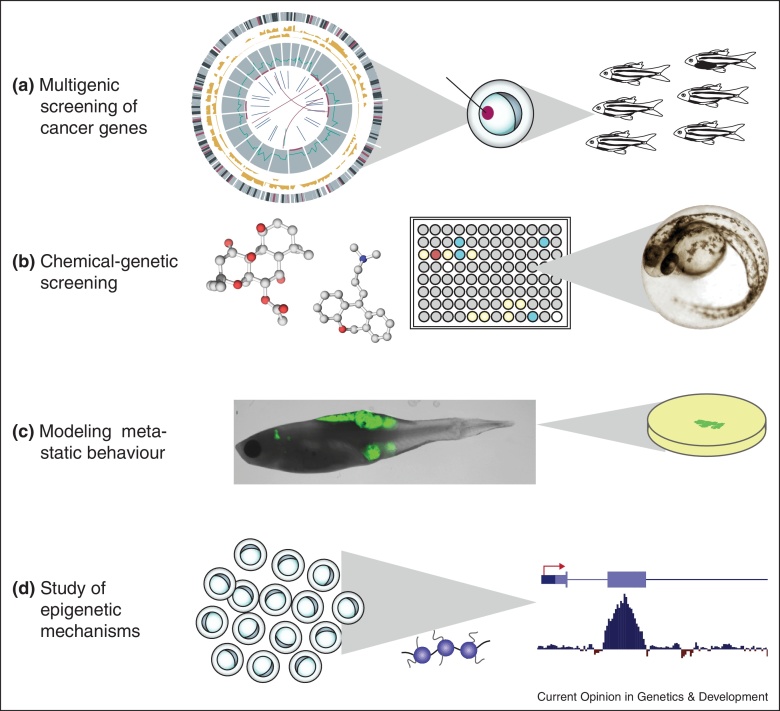
Important areas of zebrafish application in cancer research. **(a)** Multigenic screening involves the parallel testing of the oncogenic potential of candidate cancer genes by injecting plasmids harboring gene of interests into embryos and monitoring for accelerated tumor onset in adult fish [28^••^]. **(b)** Chemical libraries can be screened for activity in live zebrafish embryos using early embryonic phenotypic markers in 96 well plates. **(c)** Tumor metastasis can be followed through the injection of GFP-labelled cell cultures in transparent zebrafish, called *casper* [17]. **(d)** One method of studying cancer epigenetics in fish is to perform chromatin immunoprecipitation (ChIP) upon FACs sorted embryos followed by sequencing or expression profiling. Embryos can be rapidly collected in tens of thousands of batches using the i-spawn [55].

The zebrafish field has seen major growth over the past 10 years, as rapid application of transgenic and chemical screening techniques have placed the fish in a unique category of cancer models. But while creating and analyzing models of human cancer is useful, it ultimately is not significantly advantageous to that done in mouse models. For the fish to offer truly novel and important insights into human cancer will require major innovations in technology and scale. Several areas are particularly amenable to study in the zebrafish, as outlined below (Figure 1).

#### Multigenic changes in cancer

It is increasingly recognized that most human cancers are wildly heterogeneous at genetic, and likely, epigenetic, levels. To fully capture this complexity will require *in vivo* models that can express not just one to four altered genes, but potentially dozens. The increasing sophistication in making knockouts using TALENS [49,49] and the Cas9/CRISPr [50] genome editing system has made it possible to target nearly any candidate cancer gene in the *in vivo* setting. Although CRISPr was initially thought to be primarily useful for generating germline mutations [50,51], more recent work has highlighted its capacity for inducing somatic, biallelic disruptions in the F0 injected fish [52]. This is a tremendous advantage in zebrafish, since thousands of embryos per day can be generated, each of which can conceptually be injected with a CRISPr and phenotypes directly assessed without going to the next generation. In a typical fish facility containing 2000–10 000 adult pairs of fish, the capacity to test hundreds of candidate genes serially or in parallel dwarfs what can be achieved in mouse models. It seems likely that large-scale genetic screens using this methodology in zebrafish will be forthcoming in the near future, complementing what has been done using ENU screens.

#### Chemical screening

Traditionally it has been difficult to perform large-scale chemical screens *in vivo.* However, numerous studies have now shown that the zebrafish is highly amenable to large-scale screens, testing thousands of compounds using detailed, *in vivo* phenotypic readouts. Although the majority of these screens have relied upon ‘proxy’ embryonic phenotypes (i.e. an embryonic gene program thought related to an adult cancer phenotype), better models of young fish with *bona fide* cancer will make screening directly in young fish possible.

#### Modeling metastasis

Responsible for nearly all deaths from solid tumors, the capacity to accurately model metastasis *in vivo* is essential to improving cancer survival. Our group (RW) has developed a transparent adult zebrafish, *casper*, that offers very high sensitivity for imaging each of the steps of metastasis [53]. Combining the optical superiority of this model with all of the other key technologies (transgenesis, transplantation, chemical screens, CRISPr's), and with the pool of available mutants generated from the Zebrafish Mutation Project [54], the zebrafish offers a completely unique model in which to deeply probe the biology of metastasis.

#### Epigenetics changes in cancer

A few studies (e.g. the discovery of SETDB1 in melanoma [28^••^] as mentioned above) have just begun to explore how the zebrafish can be used to understand epigenetic contributions to cancer. This clearly emerging field will greatly benefit from the genetic and chemical screening tools available in the fish. Improvements in performing core biochemical techniques (i.e. ChIP-seq, methyl-seq, RNA-seq) along with zebrafish cell lines and antibodies will potentially allow for probing of how epigenetic changes contribute to cancer phenotypes. Rapid and large-scale transgenesis, particularly with inducible systems, will be a key method to determine the temporal dynamics of such changes, which will differ from purely genetic changes seen in many tumor types.

## Conclusion

As we enter the post-genomics era, the stage is set for zebrafish researchers to capitalize on the strengths of this model system and make significant contributions to cancer research. Already, zebrafish have shown great potential through proof-of-principle experiments involving high-throughput screening [18^••^,23^•^,28^••^,30^••^] and detailed live imaging [17,31,33,34] of embryonic and adult phenotypes. New genomic technologies have provided greater resolution for performing analyses of zebrafish cancer but require careful application and interpretation. In order to fully maximize the potential of zebrafish in cancer research, strategic areas, such as systematic and scalable methods of functional gene interrogation, using the multitude of existing models, should become a priority. Such focused efforts will inevitably lead zebrafish toward an impact on cancer research that is far more vital and productive.

## References and recommended reading

Papers of particular interest, published within the period of review, have been highlighted as:

• of special interest
•• of outstanding interest

## Figures and Tables

**Table 1:** Tools for modeling cancer in zebrafish.

Purpose	Tool	Examples
Mutagenesis for forward genetic screens	*N*-ethyl-*N*-nitrosourea (ENU) [56]	[3^•^,5,57]
	Retroviral-based insertional mutagenesis [58]	[59]

Transgenesis	Tol2 transposon [60,61]	[16]

Transgenesis for inducible gene expression	GAL4/UAS [62]	[63]
	Heat-shock Cre/loxP [64,65]	[11,20,66,67]
	Tet-on [68]	[69]
	LexPR system [70]	[14]

Site-specific mutagenesis	Talens [71]	[72]
	Zinc finger nucleases [73,74]	[75]
	CRISPr [50]	[52]

Selective expression of mutant alleles in somatic tissue	Plasmid injection (miniCoopR shuttle vector system) [28^••^]	[28]
